# Immune Evasive Effects of SARS-CoV-2 Variants to COVID-19 Emergency Used Vaccines

**DOI:** 10.3389/fimmu.2021.771242

**Published:** 2021-11-22

**Authors:** Yandi Zhang, Jo-Lewis Banga Ndzouboukou, Mengze Gan, Xiaosong Lin, Xionglin Fan

**Affiliations:** Department of Pathogen Biology, School of Basic Medicine, Tongji Medical College, Huazhong University of Science and Technology, Wuhan, China

**Keywords:** COVID-19, SARS-CoV-2 variants, vaccine efficacy, immune evasive, epidemiology

## Abstract

Coronavirus disease 2019 (COVID-19) pandemic is a serious threat to global public health and social and economic development. Various vaccine platforms have been developed rapidly and unprecedentedly, and at least 16 vaccines receive emergency use authorization (EUA). However, the causative pathogen severe acute respiratory syndrome coronavirus-2 (SARS-CoV-2) has continued to evolve and mutate, emerging lots of viral variants. Several variants have successfully become the predominant strains and spread all over the world because of their ability to evade the pre-existing immunity obtained after previous infections with prototype strain or immunizations. Here, we summarized the prevalence and biological structure of these variants and the efficacy of currently used vaccines against the SARS-CoV-2 variants to provide guidance on how to design vaccines more rationally against the variants.

## Introduction

Severe acute respiratory syndrome coronavirus-2 (SARS-CoV-2), the causative pathogen of coronavirus disease 2019 (COVID-19), was first reported in early December 2019 in Wuhan, China (1). As of 18, October 2021, the COVID-19 pandemic has resulted in more than 240 million confirmed cases worldwide, with at least 4.9 million deaths (2). To deal with the ongoing COVID-19 pandemic, a variety of vaccines have been developed rapidly and unprecedentedly. Various vaccine platforms are available for the development of SARS-CoV-2 vaccines, such as protein or peptide subunit, recombinant viral vectors, nucleic acid (DNA or mRNA)-based vaccines, live attenuated virus, inactivated whole virus, and virus-like particle, etc. There are 126 vaccines currently in clinical development and 194 vaccine candidates at the pre-clinical developmental stage (3). More importantly, at least 16 vaccines have now gained EUA from their National Regulators Authorities (NRAs) of different countries. Globally, as of 18, October 2021, a cumulative number of 6 billion vaccine doses have been administered (2). Interestingly, clinical trial results showed that all of them could provide good efficacies to protect immunized individuals against infection with SARS-CoV-2 prototypical, wild-type SARS-CoV-2 Wuhan strain, especially to arrest the disease progression and severity in real and natural environments.

However, with the number of infected individuals increasing and the epidemic continuing, SARS-CoV-2 has been found to continue to evolve and mutate, with many viral variants emerging in different regions of countries. A few variants have successfully evolved and become the predominant strains to replace the prototype strain under selective pressure and then spread out across the world with the pandemic (4). Correspondingly, a key issue has been raised whether the currently available emergency vaccines could effectively protect against these emerging epidemic variants.

**Figure 1:**
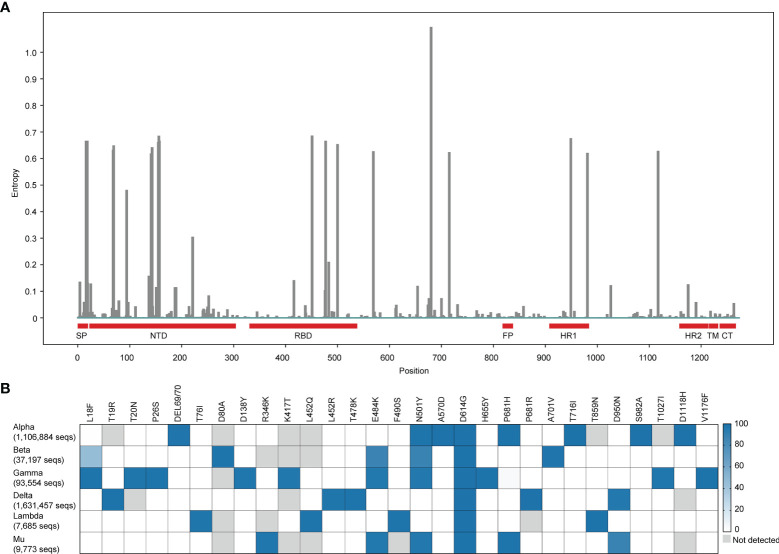
Global mutation landscape of spike and mutation prevalence across lineages in variants of concern or interest. **(A)** Global mutation landscape of spike. Based on a spike alignment containing 3,105,458 sequences around the world (https://cov.lanl.gov, accessed 3 October 2021.), the frequencies of mutations on the different positions of the spike protein of SARS-COV-2 were analyzed and shown in the figure. **(B)** Mutation prevalence across lineages in variants of concern or interest. We select the mutations with > 95% prevalence in at least one lineage to product the mutation prevalence across lineages of concern or interest, using outbreak lineage comparison (https://outbreak.info/compare-lineages, accessed 13 October 2021) in 4,204,861 sequences. The color of blue means the higher mutation frequency. The grey color means no mutations detected.

The SARS-CoV-2 uses the angiotensin-converting enzyme 2 (ACE2), the same receptor as SARS-CoV (5), to infect humans. The spike (S) protein on the viral envelope of SARS-CoV-2 with a total length of 1,273 amino acids recognizes and binds to the ACE2 receptor on the host cell. S protein contains five functional domains. N-terminal domain (NTD, 12–306 residues) and receptor-binding domain (RBD, 328-533 residues) on S1 subunit are responsible for receptor binding. The fusion peptide (FP, 815-826 residues), heptapeptide repeat sequence 1 (HR1, 912–984 residues), and HR2 (1163–1213 residues) on S2 subunit are involved in the process of membrane fusion (6). The earliest reported SARS-CoV-2 variant had a D614G mutant on the S protein in April 2020 (7). Since then, the emergency of SARS-CoV-2 variants has attracted significant attention. If SARS-CoV-2 variants show an effective adaptation to a new host, they might have positive genetic mutations that affect receptor binding and enhance multiplication and propagation of the virus in infected people (8). Up to now, more than 1200 amino acid sites of the S protein have been found capable to produce mutations with the genome of Wuhan-Hu-1 (GenBank: NC_045512) as a reference sequence (**Figure 1A**). Different countries have reported a large of SARS-CoV-2 new variants. These mutations were mainly concentrated on RBD, NTD, and HR1 of the S protein. To deeply understand the impact of these mutations on the transmission capacity and virulence of SARS-CoV-2, we elaborated on mutations and epidemiology of SARS-CoV-2 variants and their evasive immune effects to different emergency used vaccines. Our works provide a scientific background for designing and developing more effective vaccines against SARS-CoV-2 variants.

## Classification and Epidemiology of SARS-CoV-2 Variants

For unification and standardization, WHO, recently, reclassified SARS-CoV-2 variants as variants of concern (VOC) and variants of interest (VOI) emerging around the world, based on their prevalence, transmissibility, disease severity, and immune escape ability (**Table 1**) (15). VOC consist of the following variants. Alpha as known as B.1.1.7 and new Q sub-lineages in Pango lineage (9), 20I (V1) in next strain clade, GRY (formerly GR/501Y.V1) in GISAID clade, was first identified in United Kingdom, in September 2020. Beta, known as B.1.351 in Pango lineage (10), 20H (V2) in nextstrain clade, GH/501Y.V2 in GISAID clade, was first identified in South Africa, in May 2020. Gamma, known as P.1 in Pango lineage (11), 20J (V3) in nextstrain clade, GR/501Y.V3 in GISAID clade, was first identified in Brazil, in November 2020. Delta, known as B.1.617.2 and new AY sub-lineages in Pango lineage (12), 21A in next strain clade, G/478K.V1 in GISAID clade, was first identified in India, in October 2020.

**Table 1:** The mutations on the spike of VOC and VOI.

WHO classification	Variants	Mutations in spike	Reference
VOC	B.1.1.7 (Alpha)	69-70 del, 144 del, N501Y, A570D, D614G, P681H, T716I, S982A, D1118H.	(9)
	B.1.351 (Beta)	L18F, D80A, D215G, 242-244 del, R246I, K417N, E484K, N501Y, D614G, A701V.	(10)
	P.1 (Gamma)	L18F, T20N, P26S, D138Y, R190S, K417T, E484K, N501Y, D614G, H655Y, T1027I, V1176F.	(11)
	B.1.617.2 (Delta)	T19R, (V70F*), T95I, G142D, E156-, F157-, R158G, (A222V*), (W258L*), (K417N*), L452R, T478K, D614G, P681R, D950N.	(12)
VOI	C.37 (Lambda)	G75V, T76I, del RSYLTPGD246-253N, L452Q, F490S, D614G, T859N.	(13)
	B.1.621 (Mu)	T95I, Y144T, Y145S, ins146N, R346K, E484K, N501Y, D614G, P681H, D950N.	(14)

VOC, Variants of concern; VOI, Interested variants.

All mutation sites are based on Wuhan-Hu-1 (GenBank: NC_045512) as the reference sequence.

*detected in some sequences but not all.

**Figure 2:**
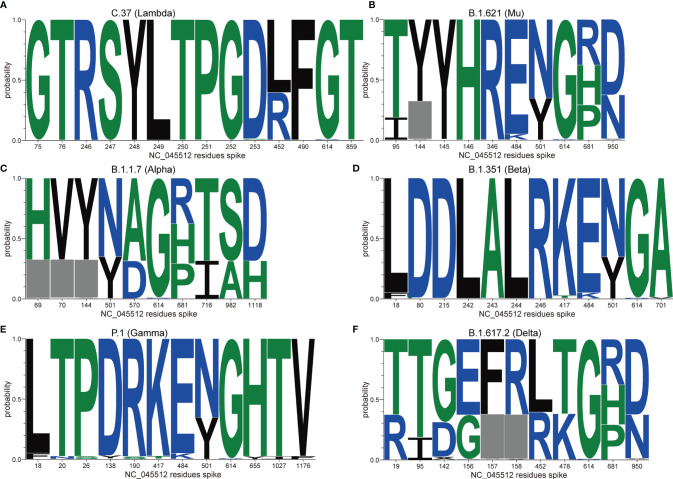
Frequencies of VOC and VOI in relevant positions. We collect and analyze the frequencies for the spike mutations in VOI **(A, B)** and VOC **(C–F)** worldwide from December 24, 2019 to October 13, 2021, using the Analyze Align (AA) tool at cov.lanl.gov (https://cov.lanl.gov/content/index). The log on the vertical axis indicates the amino acid frequency in the data set and the horizontal axis shows the mutation sites in the S protein, with NC_045512 as a reference. The gray box indicates deletion.

VOI contain Lambda (a.k.a., C.37) from Peru (13) and Mu (a.k.a., B.1.621) from Colombia (14). Lambda with an emerging mutation(F490S) in RBD and a deletion (at positions 247/253) in NTD has been detected in at least 33 countries (**Figure 2A**) (16). Mu with an emerging mutation (R346K) in RBD and a P681H mutation in the furin cleavage site has been detected in at least 49 countries (**Figure 2B**) (17). In addition, several variants such as B.1.427/429, B.1.525, B.1.526, and B.1.617.1, previously designated as VOI have finally proven to no longer pose a significant risk to global public health (15).

Interestingly, we observed there were 3,121,316 sequences containing the G614 mutation in a total of 3,148,973 spike sequences (ratio, 0.99) from cov.lanl.gov (https://cov.lanl.gov/content/index) and found that the first discovered mutation D614G existed in almost all variants. This mutation benefits the open state of the homologous S protein trimer, which can help the S protein to bind closely with the receptor and thus promoting infection and transmission (18). Moreover, there are many combinations of mutations in the RBD among VOC. These usually occur in several key positions across different VOC lineages, such as K417, L452, T478, E484, and N501, P681 etc. (**Figure 1B**). Importantly, convergent evolution of mutations confers to the stronger ability of VOC for either transmission or immune evasion (19).

**Figure 3:**
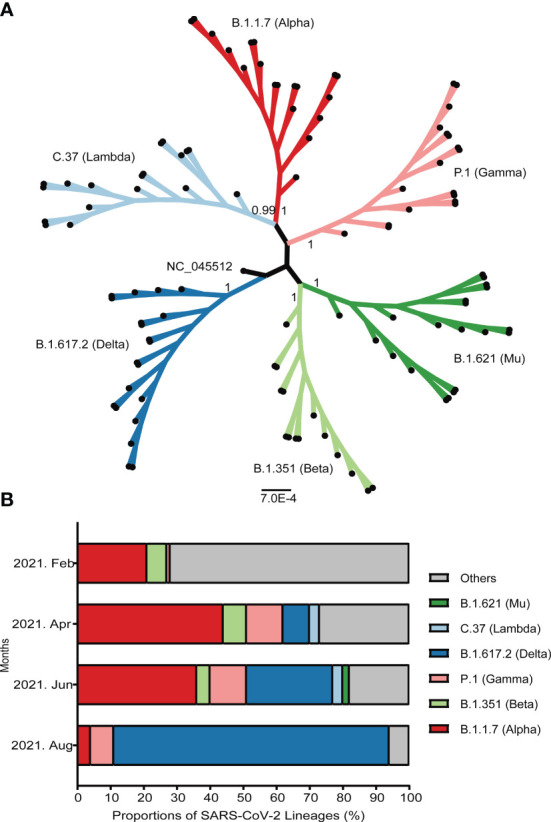
Emergence and prevalence of several important SARS-CoV-2 variants around the world. **(A)** The maximum-likelihood phylogenetic tree of SARS-CoV-2. The bootstrap values were shown at the corresponding nodes. The branches with dots at the ends represented randomly selected sequences. **(B)** The prevalence of several important variants from February to August, 2021. The ordinate represented the proportion of different linages. The strains were shown by different colors that marked on the map.

Since April 2021, VOC have accounted for more than 70% of all widespread variants globally. Among these, B.1.1.7 has attributed nearly half of all detected strains. Another prevalent strain belongs to B.1.351 lineage, which remained stable at around 7% of all detected strains. The epidemic variants B.1.617.2, identified Indian strain and P.1, earlier reported Brazilian strain have been increasing rapidly. The detected proportion of B.1.617.2 has rose to over 80% in August 2021 (**Figure 3**).

### G614 Variant

Korber et al. first reported that the variant with the D614G mutation on the S protein emerged during early COVID19 pandemic (20). Since then, it has rapidly become the predominant circulating variant. At the same time, a study showed that D614G variant infections resulted in a higher level of viral load and became more infectious or transmissible than D614 wild type virus did, although no evidence supported that D614G variant was associated with high mortality or disease severity (21). This finding was consistent with the results from animal challenge experiments (22). Interestingly, antibodies induced by either G614 variant or the prototype strain infections have cross-neutralization activity (21, 22).

### B.1.1.7 Lineage

B.1.1.7 lineage, also known as VOC-202012/01, or 20B/501Y.V1, first emerged in September 2020 (23, 24) and became the predominant strain in UK quickly. It has been detected in at least 180 countries (25). B.1.1.7 contains 7 amino acid substitutions in the S protein (**Figure 2C**), a N501Y mutation in the RBD (i.e., an amino acid substitution of asparagine to tyrosine at position 501) and 3 amino acids deletions in the viral NTD (i.e., the deletions of histidine, valine and tyrosine at positions 69-70 and 144/145, respectively) (26). Another mutation P681H is adjacent to the furin cleavage site which plays an essential role in viral transmission and infection (27). Thus, B.1.1.7 may be more transmissible and possibly stronger pathogenicity than other SARS-CoV-2 variants (28). Even so, neutralization antibody (nAb) titers of sera from immunized individuals or convalescent patients against B.1.1.7 lineage only slightly decreased, based on the pseudovirus neutralization assay (29).

### B.1.315 Lineage

B.1.351 lineage (a.k.a., Beta or 20H/501Y.V2) first identified in South Africa has been detected in at least 114 countries and 51 states of USA (30). During December 2020 to March 2021, the average daily prevalence of B.1.351 in South African reached up to 80% (30). B.1.351 lineage contains 9 amino acid substitutions in the S protein, such as K417N, E484K, and N501Y in the viral RBD, and 3 amino acids deleted at positions 242-244 in the viral NTD (10). The combination of three mutations K417N/E484K/N501Y in the RBD region resulted in a significant decrease of anti-RBD nAb titers. Of these mutations, E484K plays a major role (31). K417N reduces the neutralizing activity through attenuating its polar contact with the complementary determination region (31). The 242-244 deletion reduces the neutralizing activity of anti-NTD nAb *via* changing the conformation of the NTD antigen supersite (**Figure 2D**) (32, 33). More importantly, B.1.351 variants showed significant resistance to the nAb in the sera of recovered patients and immunized population (34). Interestingly, the serum of convalescent patients infected with the B.1.351 variant showed effective cross-neutralization against other variants (35).

### P.1 Lineage

P.1 lineage (a.k.a., Gamma) was first identified in Brazil with several mutations of biological significance such as E484K, N501Y and K417T in the RBD and five mutations in the NTD (**Figure 2E**) (32, 33). Currently, P.1 has been detected in at least 75 countries. From April to June 2021, 80% daily infected individuals in Brazil were caused by P.1 (36). The study demonstrated that the infectivity of P.1 might be 1.7 to 2.4 times higher than non-P.1 infections (37). Moreover, the sera from immunized individuals or convalescent plasma showed a more significant drop of neutralizing activity against P.1 (38).

### B.1.617.2 Lineage

B.1.617.2 (a.k.a., Delta), reclassified by WHO as VOC due to its high transmissibility and pathogenicity, has been detected in at least 157 countries since its outbreak in April 2021. More than 90% cases per day were caused by B.1.617.2 in India (39). It contains 8 amino acid substitutions in the spike protein and 2 amino deletions, i.e., L452R and T478K mutations in RBD along with a P681R in the furin cleavage site (**Figure 2F**) (40). These mutations not only enhance the ability of B.1.617.2 to bind with the ACE2 receptor, but also improve the cutting efficiency between S1 and S2 units. Eventually, the variant is highly contagious, with a stronger transmission and a higher risk of hospitalization, when compared to B.1.1.7 (41). It is noteworthy that seropositive individuals vaccinated current available vaccines could not provide sufficient protection to prevent B.1.617.2 infections (42, 43).

## Efficacy of COVID-19 Vaccines Against SARS-CoV-2 Variants

Here, we have examined all the vaccines having received EUA from RNAs (**Table 2**) and their protective efficacies on the prototype strain of SARS-CoV-2 and its various variants (**Table 3**).

**Table 2:** A summary of all vaccines received EUA from NRAs.

Vaccine platform	Manufacturer	Name of Vaccine	Vaccine antigen design	Delivery system	Doses/Schedule/Route	stage	WHO recommendation	Reference
RNA based vaccine	Pfizer-BioNTech-Fosun Pharma	COMIRNATY^®^(BNT162b2)	mRNA encoding the full-length S-2P protein	LNP	2/day 0 + 21/im	Phase 4	Yes	(44)
	Moderna	Spikevax (mRNA-1273)	mRNA encoding the full-length S-2 P protein	LNP	2/day 0 + 28/im	Phase 4	Yes	(45)
Viral vector	AstraZeneca-University of Oxford	Vaxzevria/AZD1222 (ChAdOx1 nCoV-19)	tPA leader and the full-length S gene	ChAdOx1 (Non-replicating)	1-2/day 0 + 28/im	Phase 4	Yes	(46)
	Serum Institute of India Pvt. Ltd	Covishield (ChAdOx1_nCoV-19)	tPA leader and the full-length S gene	ChAdOx1 (Non-replicating)	1-2/day 0 + 28/im	Phase 4	Yes	(46)
	Janssen	Ad26.COV2.S	Furin cleavage site mutation and the gene for S-2P protein	rAd26, (Non-replicating)	1-2/day 0 or day 0 + 56/im	Phase 4	Yes	(47)
	The Gamaleya national center	Sputnik V	Full-length S gene	rAd5 and rAd26	2/day 0 + 21/im	Phase 3	No	(48)
	CanSino	Convidecia (Ad5-nCoV)	tPA leader and the full-length S gene	rAd5, (Non-replicating)	1/day 0/im	Phase 4	No	(49)
Inactivated virus	Sinopharm/BIBP	BBIBP-CorV	Inactivated SARS-CoV-2 (HB02)	Aluminum hydroxide	2/day 0 + 21/im	Phase 4	Yes	(50)
	Sinopharm/WIBP	SARS-CoV-2 Vaccine (Vero Cell), Inactivated (lnCoV)	Inactivated SARS-CoV-2 (WIV04)	Aluminum hydroxide	2/day 0 + 21/im	Phase 3	No	(51)
	Sinovac	CoronaVac	Inactivated SARS-CoV-2	Aluminum hydroxide	2/day 0 + 14/im	Phase 4	Yes	(52)
	Bharat Biotech, India	COVAXIN (BBV152)	Inactivated SARS-CoV-2 (NIV-2020-770)	Algel and IMDG	2/day 0 + 14/im	Phase 3	No	(53)
Recombinant protein	Zhifei Longcom, China	ZF2001	Dimeric RBD	Aluminum hydroxide	3/day 0 + 28 + 56/im	Phase 3	No	(54)
	BioCubaFarma-Instituto Finlay de Vacunas	Soberana 02 +Soberana Plus	Monomeric RBD	Tetanus toxoid	3(Soberana 02+ Soberana 02+ Soberana Plus)/day 0 + 28+56/im	Phase 3	No	(55, 56)
	Center for Genetic engineering and biothechnology	Abdala	Monomeric RBD	Aluminum hydroxide	day 0 + 28+56 (0 + 14+28)/im	Phase 3	No	(55)
	Novavax	Covovax (NVX-CoV2373)	Furin cleavage site mutation and S-2P protein	Matrix-M1 adjuvant	2/day 0 + 21/im	Phase 3	No	(57)
Peptide antigen	Vector State Research Centre of Virology and Biotechnology	EpiVacCorona	N protein and peptides from S protein	Aluminum hydroxide	2/day 0 + 21/im	Phase 3	No	(58)
DNA based vaccine	Zydus Cadila	ZyCoV-D	DNA coding S protein and IgE signal peptide	NFIS	3/day 0 + 28+56/NFIS	Phase 3	No	(59, 60)

EUA, emergency use authorization; NRAs, National Regulators Authorities; LNP, lipid nanoparticle; ChAdOx1, chimpanzee adenovirus; rAd5, adenovirus type-5-vector; rAd26, adenovirus type-26-vector; Algel, Aluminum hydroxide; IMDG, Imidazoquinoline; NFIS, needle free injection system.

**Table 3:** The efficacy of all vaccines received EUA against SARS-CoV-2 variants.

Vaccine	Overall VE	VE for variants				Reference
		B.1.1.7	B.1.351	P.1	B.1.617.2	
BNT162b2	95%	93%	75.0%	61% (single dose)**^a^**	88%	(41, 61–63)
Spikevax (mRNA-1273)	94.1%	100%	96.4%	95.5%	86.7%	(64–66)
Sinopharm (BIBP)	72.8%	NA	NA	NA	59%**^c^**	(51, 67)
Sinopharm (WIBP)	78.1%	NA	NA	NA	59%**^c^**	(51, 67)
CoronaVac	50.7% (Brazil)	NA	NA	50%**^b^**	59%**^c^**	(67–70)
	83.5% (Turkey)					
	65.9% (Chile)					
AZD1222/ChAdOx1 nCoV-19	66.7%	70.4%	10.4%	64%	67%	(42, 71–74)
Ad26.COV2.S	66.1% (moderate to severe)	NA	64% (moderate to severe)	68% (moderate to severe)	NA	(75–77)
	85.4% (severe to critical)		81.7% (severe to critical)	88% (severe to critical) **^b^**		
Sputnik V	91.6%	NA	NA	NA	NA	(48)
Convidecia (Ad5-nCoV)	65%-69% (overall)	NA	NA	NA	NA	(78)
	90%-96% (severe)					
ZF2001	81.76%	92.93%	NA	NA	77.54%	(79)
EpiVacCorona	NA	NA	NA	NA	NA	(58)
COVAXIN (BBV152)	77.8%	NA	NA	NA	65.2%	(80)
ZyCoV-D	67%	NA	NA	NA	67%**^b^**	(59)
Abdala	92.28%	NA	NA	NA	NA	(81)
Soberana02+plus	91.2%	NA	NA	NA	91.7%	(81)
Covovax (NVX-CoV2373)	89.7%	85.6%	51%	NA	NA	(82, 83)

VE, vaccine efficacy; BIBP, Beijing Institute of Biological Products Co-Ltd; WIBP, Wuhan Institute of Biological Products Co-Ltd; NA, not available.

All data regarding the vaccine efficacy against a single variant, respectively, are obtained after vaccination according to the recommended vaccination schedule. Unless otherwise specified introduction. The data shows a possible trend in the impact of variants on vaccines, owing to differences in efficacy end points.

^a^Single dose vaccination, no data of recommended vaccination dose and schedule.

^b^A real-world vaccine efficacy is estimated in the areas where variants is widespread.

^c^Vaccine efficacy of China’s three inactivated SARS-CoV-2 from Sinopharm and Sinovac vaccines against the Delta variant.

### BNT162b2 mRNA Vaccine

Since December 2, 2020, BNT162b2 (Pfizer – BioNTech – Fosun Pharma) was used as an emergency vaccine in the UK for the first time to prevent people over 16 years of age against the infection with COVID-19 (84). Much later, the U.S. FDA approved the emergency use of the Pfizer vaccine (85). BNT162b2 was constructed based on an mRNA modified by nucleosides with substitution of two prolines encoding the complete S protein (S-2P) of the prototype strain SARS-CoV-2 and encapsulated in a nanoparticle lipid (LNP) which aims to protect mRNA from the degradation (44). A phase 2/3 clinical trial result showed that BNT162b2 conferred almost 95% protection against SARS-CoV-2 infection after the second dose (61). Other studies showed that the vaccine efficacies were approximately 88%, 93%, and 75.0% against the Delta, Alpha, and Beta variants, respectively (41, 62). Skowronski et al. conducted a clinical study in British Columbia and Canada, and found that the vaccine was 61% effective against Gamma variants at ≥21 days after individuals received the first dose of the vaccine (63). BNT162b2 vaccination effectively reduced the risk of hospitalization of patients infected with SARS-CoV-2 prototype strain, as well as the Delta strain (42). Although the protection against variant B.1.351 significantly reduced, antibodies induced by BNT162b2 showed effective against variants B.1.1.7, COH.20G/677H Columbus Ohio, and 20A.EU2 Europe (86). Interestingly, the effect of vaccination might be affected by several factors. Among these, age might be a key factor. Collier et al. showed a significant drop of serum nAb titers with age in immunized individuals, and the protection against variants B.1.1.7, B.1.351, and P.1 also decreased. Moreover, the number of SARS-CoV-2 specific memory B cells, IFN-γ^+^ and/or IL-2^+^ T cells was lower in vaccinated people over 80 years old than in younger people (87). Besides, BNT162b2 vaccine indicated high efficacy in pregnant women and immunized adolescents aged 12 to 15 years, and the efficacy was similar to that in the general population (88, 89). Interestingly, in a study in the UK, antibody responses to BNT162b2 showed that women and the individuals young and previously infected with SARS-CoV-2 had higher IgG levels afterward vaccination (90). However, whether gender is the factor to affect the immunization remains controversial (90, 91). In immunocompromised individuals receiving immunosuppressive drugs, Hadjadj et al. observed that antibody responses had partial neutralizing activity against Alpha, and even weaker against Delta stains, and their T cell responses were also altered (92). Therefore, immunosuppressive drugs for specific diseases might affect the vaccine-induced immunological effects. However, in patients with solid malignancies, the nAb (seroconversion: 79% in a vaccine, 84% in control) and polyfunctional T cell responses could be observed six months after vaccination (93).

### Moderna mRNA-1273

Another mRNA-based vaccine, known as mRNA-1273, was developed by Moderna and the National Institute of Allergy and Infectious Diseases. It was prepared by using mRNA, which encoded the prefusion stabilized S-2P protein, then encapsulated in a lipid nanoparticle (45). The mRNA-1273 vaccine was approved for emergency use by the FDA on December 18, 2020 (94). A phase 3 clinical trial demonstrated 94.1% efficacy of mRNA-1273 vaccine against COVID-19 illnesses, including serious illnesses, in participants aged ≥18 years (64). Also, the mRNA-1273 showed highly protective efficacies against variants B.1.1.7 and B.1.351 infection in all cases of COVID-19, i.e., 88.1% and 61.3% ≥ 14 days post-one dose, 100% and 96.4% post-two doses (65). Furthermore, the mRNA-1273 vaccine remained highly effective against Gamma and emerging Delta and Mu variants , and the vaccine efficacies against these variants were 95.5%, 86.7% and 90.4%, respectively. However, the efficacies against these variants gradually decreased with the duration of the post-vaccination time (66). On the other hand, the vaccine-induced antibodies showed neutralizing activity against four variants, including nCoV/USA_WA1/2020 (line A.1), EHC-083E (line B.1), B.1.1.7, and N501Y (95), but the neutralization activity against B.1.351 variant was significantly lost (96). Remarkably, S protein-specific IFN-γ+ T lymphocyte responses against B.1.1.7 and B.1.351 variants were induced after immunization despite reduced nAb titers (97). Unlike another mRNA vaccine, BNT162b2, similar levels of nAb were observed in mRNA-1273 vaccinated adolescents aged 12 to 15 years, in people aged 56 years and older, and in pregnant women (98, 99). Besides, the immune status of the body also affected vaccine efficacy. A study of immunocompromised populations showed seropositivity rates of 98.1% for healthcare workers, 37.2% for solid organ transplants, 83.8% for autoimmune diseases, 54.7% for hematologic malignancies and 82.4% for solid tumors (100). In addition to the above, Moderna developed another vaccine against the B.1.351 and other variants, called mRNA-1273.351, based on cross-neutralizing activity (101).

### Sinopharm Inactivated SARS-CoV-2 Vaccine

The inactivated SARS-CoV-2 (lnCoV) vaccine was produced by the Sinopharm-Beijing Bio-Institute of Biological Products (BIBP) and the Sinopharm-Wuhan Institute of Biological Products (WIBP) received the EUA. Aluminum hydroxide adjuvanted vaccine was prepared by culturing viruses in infected Vero cell lines, then inactivating viruses (HB02 and WIV04) with β-propiolactone and chromatography purification (50, 51). Phase 3 clinical studies reported 72.8% and 78.1% vaccine efficacies and 94.5 and 156.0 geometric mean titers (GMTs) at day 14 after the second dose for Sinopharm-WBIP and Sinopharm-BBIP, respectively, that were effective against prototype SARS-CoV-2 infection (51). Besides, Li et al. showed that the overall efficacy after vaccination was 59.0% against SARS-CoV-2, 70.2% against COVID -19 moderate and nearly 100% against severe disease during the Delta variant outbreak in Guangzhou city, China (67). Although, sera from recipients BBIBP-CorV vaccine showed neutralizing activity against the variant B.1.1.7 and the prototype strain, most sera samples showed complete or partial loss of the neutralization against B.1.351 lineage (102, 103). Remarkably, similar to BNT162b2, the influence of gender on immune response is also controversial. One study showed significantly higher vaccine efficacy in women than in men during the Delta variant epidemic (67), but another study showed gender had no effect on IgM and IgG antibodies responses (104). However, age can affect the IgG antibody response, and the antibody level of elderly recipients (≥42 years old) is significantly lower than that of younger (104).

### CoronaVac From Sinovac

Another inactivated vaccine against SARS-CoV-2 was developed by Sinovac Biotech Company *via* a bioreactor (52). This inactivated vaccine was cleared for emergency use in July 2020 and subsequently approved by the WHO (105). A phase 3 clinical trial reported that the vaccine efficacies were 50.7% in Brazil (68), 83.5% in Turkey (69), and 65.9% in Chile (70), respectively. However, it was highly effective against severe forms, hospitalizations and deaths of COVID-19 (106). In particular, vaccine-induced serum nAb remained effective against D614G, B.1.1.7 and B.1.429 variants (107), but that was significantly less effective against B.1.351 (107), P.1 and P.2 variants (68). Interestingly, although an IFN-γ-secreted T-cell response and a B-cell response were significantly induced in a Chilean study, no differences were observed between the age of 18-59 and over 60 (70). Nevertheless, CoronaVac was able to induce a strong nAb response (GMTs: 86.4-142.2) in children and adolescents aged from 3 to 17 years (108). In order to study the response of specific immune populations to the CoronaVac vaccine, Sinovac conducted a phase 4 clinical trial, which observed a reduction more than 15% in seroconversion rates and nAb titers after the second dose compared to controls (109).

### AZD1222/ChAdOx1 nCoV-19 From AstraZeneca

The University of Oxford, in collaboration with the British company AstraZeneca and the Serum Institute of India, developed the AZD1222 vaccine, which was prepared using a chimpanzee adenovirus deficient in the replication ChAdOx1 to express the S protein of SARS -CoV-2 (46). The WHO has approved the AZD-1222 vaccine for the prevention of COVID-19 (110). However, several side effects, such as disseminated intravascular coagulation and brain blood vessels have been reported in people who received this vaccine (111). Some EU countries suspended the use of this vaccine. After carefully evaluating the available data generated by the vaccine, WHO recommended continuing vaccination in a bulletin issued on March 17, 2021 (112). Approximately 14 days after the second dose, the AZD-1222 vaccine showed 66.7% efficacy against the SARS-CoV-2 prototype stain (71), 70.4% against Alpha (72), 10.4% against Beta (73), 66% against Delta (42). Although the number of cases to analyze was limited, ChAdOx1 nCoV-19 showed 64% efficacy against Gamma variant infection (74). These results indicated that the vaccine lost its protective effect against the Beta variant, but still was effective against other VOC. On the other hand, a study that vaccination was more effective in individuals aged 18 to 34 than in those aged 35 to 64 years, also in those showing signs of the prior SARS-CoV-2 infection than in those no infection. These results were confirmed by a study in populations vaccinated with ChAdOx1 or BNT162b2 SARS-CoV-2 vaccine (90, 113). However, only 28.6% of immunosuppressed patients had detectable immune responses in humoral or T-cell responses after the first administration of ChAdOx1 nCoV-19 vaccine (114).

### Ad26.COV2.S From Janssen

Ad26.COV2.S was a vaccine-based recombinant non-replicating adenoviral vector type 26 (rAd26) expressing a stabilized SARS-CoV-2 S-2P protein with a furin cleavage site mutation, developed by the Center for Virology and Vaccine Research, Harvard Medical School, collaborating with Janssen Vaccines & Prevention, Leiden (47). Studies conducted by Sadoff et al. followed by Johnson & Johnson, reported that approximately 28 days after administration, the overall vaccine efficacies were 66.1% and 85.4% for the prevention of moderate to severe and severe COVID19 diseases, respectively (75, 76). The similar vaccine efficacy was observed for variant B.1.351, i.e., 64.0% for moderate to severe COVID-19 cases and 81.7% for severe critical COVID-19 cases (75). Also, phase 3 clinical trial study showed the efficacies of Ad26.COV2.S were 68% against moderate to severe COVID-19 and 88% against severe or critical COVID-19 in the real world of Brazil, however, this effectiveness was lower in older participants than younger participants (77). Remarkably, the humoral and cellular immunity levels peaked 71 days after vaccination and could be maintained for at least 8 months. Moreover, as the time after vaccination increases, Ad26.COV2.S showed higher and broader neutralizing ability against all VOC (115). It was also noteworthy that vaccine-induced T cell responses largely retained the ability to control SARS-CoV-2 variants, although Ad26.COV2.S-induced nAb titers were reduced against B.1.351 and P.1 variants (77).

### Sputnik V From Gamaleya

Sputnik V was a combined vaccine of a rAd26 and a recombinant adenoviral vector type 5 (rAd5) expressing an S protein (Russian Gamaleya Institute). A heterologous prime-boost regimen was utilized for the vaccine consisting of the first dose (rAd26) and the second dose (rAd5) at an interval of 21 days. An interim analysis in phase 3 clinical trial showed that the Sputnik V vaccine could induce the efficacies of 91.6% against COVID-19 and 100% against severe COVID-19 at 21 days after the first dose (48). The high levels of vaccine-induced humoral and cellular responses were similar between different age groups and between males and females, and were not affected by previous SARS-CoV-2 infections (48). In addition, in a randomized controlled study in Argentina, the participant’s sera demonstrated neutralizing activity against the SARS-CoV-2 prototype strain and B.1.1.7 variant, although a significant reduction in the GMTs was observed against the variant containing the E484K mutation such as B.1.351 variant (116).

### Ad5-nCoV From CanSino

Ad5-nCoV was a vaccine, rAd5-based vaccine that expresses an un-modified S protein, which developed by the CanSinoBIO-Beijing Institute of Biotechnology (49). According to the CanSino report, the overall efficacies were 65% to 69% and 90% to 96% for critical COVID-19 at 14 to 28 days after the first dose of Ad5-nCoV, respectively (78). The antibody response induced by Ad5-nCoV vaccine peaked on day 28 post-vaccination and the specific T-cell response peaked on 14 days post-vaccination in adults aged between 18 and 60 years (117). Based on this promising result from the first human trials, CanSino conducted further investigations in a broad range of age groups, in which showed that administration with prime-boost regimen in children and adolescents aged 6-17 years (GMTs: 1037.5) produced higher levels of immune responses than adults aged 18-55 years (GMTs: 647.2) and those aged 56 years and older (GMTs: 338.0) (118). Consistent with this study, the level of immune response to the vaccine declined significantly with age in adults aged 18 years and older, and that was severely affected by the pre-existing high anti-Ad5 immunity (119).

### ZF2001

ZF2001, a RBD-based protein subunit vaccine was developed by Chinese Academy of Sciences and Anhui Zhifei Longcom Biopharmaceutical Co. Ltd. and received its EUA. In clinical trial phase 2, the ZF2001 showed high levels of nAb titers (GMTs: 94.5 for 25 µg and 117.8 for 50 µg) and complete seroconversion rates (100%) after three doses of vaccination (54). Also, a balanced immune response of Th1 and Th2 cells was observed (54). ZF2001 vaccine-elicited nAb had higher neutralizing activity against the B.1.351 variant compared to convalescents and individuals vaccinated CoronaVac, although the neutralizing activity was reduced compared to the D614G variant (103, 120). Recently, Zhifei Longcom released the latest data from phase 3 clinical trials. Indeed, this study showed that the efficacies of the ZF2001 vaccine were 77.54% and 92.93% against Delta and Alpha infections, respectively. In addition, the efficacies of all cases of COVID-19 (81.76%) and severe forms (100%) were also high (79).

### EpiVacCorona

EpiVacCorona was an aluminum hydroxide adjuvanted peptide vaccine which was covalently bound to the N protein (Vector State Research Centre of Virology and Biotechnology). The peptides were selected from the most conserved region of protein S. The EpiVacCorona vaccine provided protective immunity and eliminated antibody-dependent enhancement (ADE), which could be induced by antibodies specific for NTDs after infection. In a phase 1/2 clinical trial, Ryzhikov et al. observed a seroconversion rate of 100% and nAb titers ranging from 1:20 to 1: 160 on day 42 after the first vaccination (58).

### COVAXIN From Bharat Biotech

COVAXIN (a.k.a BBV152) was an inactivated SARS-CoV-2 strain (NIV-2020-770) adjuvant aluminum hydroxide (Algel) -Imidazoquinoline (IMDG) or Algel developed by Bharat Biotech (53), and was approved by the Drug Controller General of India (DCGI) for emergency use. In phase 1 trial, BBV152 showed good safety and tolerance, as well as, demonstrated humoral and Th1-responsive cellular immunity (121), that persisted during the three-month follow-up (122). Moreover, the vaccine induced elevated T-cell memory responses (122). A two-dose regimen of inactivated virus adjuvant Algel-IMDG could confer protection of 65.2% against B.1.617.2 variant, followed by an overall efficacy of 77.8% (80), and that efficacy against B.1.617.2 variant was not significant differences in immune responses between the age groups under 60 and over 60 (80). BBV152 was also effectively to neutralize the Alpha variant but weakly neutralize the Beta and Delta variants (123, 124).

### ZyCoV-D From Zydus Cadila

ZyCoV-D was the first DNA vaccine developed by Zydus Research Center and Vaccine Technology Center, and Cadila Healthcare Ltd, and received the EUA to be approved for use in humans on August 20, 2021 (59). ZyCoV-D comprised an optimized plasmid DNA coding spike protein along with an IgE signal sequence and was administered on the skin by the PharmaJet^®^ Tropis^®^ Needle-Free Injection System (NFIS) (60). In phase 1 study, ZyCoV-D has shown good safety and tolerability. Besides, the cellular and humoral immune responses were observed after three doses of 2 mg vaccine administered 28 days apart, and these immune responses were maintained till day 70 (125). The Indian government reported 67% vaccine efficacy against symptomatic COVID-19 in a phase 3 clinical trial (59).

### Soberana and Abdala From Cuba

Soberana and Abdala were two vaccines constructed from recombinant RBD conjugated to tetanus toxoid or aluminum (55). These two vaccines developed in Cuba has been approved by the Cuban National Control Center for Drugs and Medical Devices (CECMED) to prevent COVID-19. CECMED reported that the Abdala and Soberana vaccines were 92.28% and 91.2% effective against COVID-19, respectively (81). A regimen three-dose, i.e., two-dose Soberana02 followed by one-dose Soberana Plus showed great protective efficacy against the Delta variant. The Instituto Finlay de Vacunas reported 91.7% protective efficacy against Delta variant after three doses (56).

### NVX-CoV2373 From Novavax

NVX-CoV2373 was a recombinant protein subunit vaccine, composed of trimeric full-length S protein and Matrix-M1 adjuvant can induce Th1 phenotype CD4+ T cell response (57), which obtained its EUA on November 1 from Indonesia. However, NVX-CoV2373 demonstrated the promising results of a phase 3 clinical trial, in which the efficacies of 89.7% against SARS-CoV-2 prototype strain and 85.6% against B.1.1.7 and 51% against B.1.351 were observed after individuals received two doses of the vaccine (82, 83). Previously, Shen et al. observed that the sera of individuals who had received NVX-CoV2373 still contained high levels of nAb against the B.1.1.7 variant (126), but these nAb levels were significantly reduced 14.5-fold against B.1.315 (127). In addition, although the NVX-CoV2373 vaccine was found to induce a weak humoral immune response in the elderly, however, seroconversion occurred almost completely, with anti-spike protein IgG seroconversion rate of 97%, and nAb seroconversion rate of 100% (128).

## Conclusion

With the COVID-19 pandemic, SARS-CoV-2 evolves persistently and the number of variants increases significantly. Except for alpha, which is only highly transmissible, the other VOC are highly transmissible, with high mortality and severity of clinical cases. Among all VOC, Beta is the most infectious and pathogenic. These variants are more prevalent among young people, but are less severe. All vaccines currently used for preventing COVID-19 are based on the SARS-CoV-2 prototype strain. Although these vaccines still have great efficacy against the earliest D614 G variant and Alpha variant, they show different efficacies to other VOC and VOI. The levels of cellular and humoral immunity induced by vaccines are influenced significantly by age, the use of immunosuppressive drugs, and a history of previously infected with SARS-CoV-2. The existing vaccine platforms can also be used to rapidly prepare new vaccines against these variants. Cross-neutralizing activities and screening key epitopes among variants may be promising strategies for developing more effective vaccines that provide a comprehensive immune response against all variants.

## Author Contributions

XF contributed to the concept, and revised the manuscript. YZ did literature retrieval, collected and analyzed data, and wrote the manuscript. J-LB did literature retrieval and wrote the manuscript. MG and XL did literature retrieval. All authors contributed to the article and approved the submitted version.

## Funding

This work was supported by grants from the Applied Basic Research Key Project of Wuhan Municipal Bureau of Science and Technology (2020020601012218) and the Fundamental Research Funds for the Central Universities (HUST COVID-19 Rapid Response Call No. 2020kfyXGYJ040).

## Conflict of Interest

The authors declare that the research was conducted in the absence of any commercial or financial relationships that could be construed as a potential conflict of interest.

## Publisher’s Note

All claims expressed in this article are solely those of the authors and do not necessarily represent those of their affiliated organizations, or those of the publisher, the editors and the reviewers. Any product that may be evaluated in this article, or claim that may be made by its manufacturer, is not guaranteed or endorsed by the publisher.
